# Delay in diagnosis and associated factors among children with cancer admitted at pediatric oncology ward, University of Gondar comprehensive specialized hospital, Ethiopia: a retrospective cross-sectional study

**DOI:** 10.1186/s12885-023-10873-8

**Published:** 2023-05-22

**Authors:** Yimenu Gardie, Mulugeta Wassie, Seid Wodajo, Mastewal Giza, Mulugeta Ayalew, Yihenew Sewale, Zelalem Feleke, Melkamu Tilahun Dessie

**Affiliations:** 1 Department of Nursing, College of health sciences, Assosa University, Assosa, Ethiopia; 2 Department of medical nursing, School of Nursing, College of Health Sciences, University of Gondar, Gondar, Ethiopia; 3 Department of Midwifery, College of health sciences, Assosa University, Assosa, Ethiopia; 4 Department of public health, College of health sciences, Woldia University, Woldia, Ethiopia; 5 Department of Pediatrics, School of Medicine, University of Gondar, Gondar, Ethiopia; 6 Department of nursing, College of health sciences, Debre Berhan University, Debre Berhan, Ethiopia; 7 Department of pediatrics and child health nursing, School of Nursing, College of Health Sciences, University of Gondar, Gondar, Ethiopia

**Keywords:** Delay in diagnosis, cancer, Children, Gondar, Ethiopia

## Abstract

**Background:**

Delay in the diagnosis of childhood cancer is one of the major health problem that contribute to decreased survival rates of children particularly in developing nations. Despite advances in the field of pediatric oncology, cancer remains a leading cause of death in children. Diagnosis of childhood cancer as early as possible is crucial to reduce mortality. Therefore, the aim of this study was to assess delay in diagnosis and associated factors among children with cancer admitted to pediatric oncology ward, University of Gondar comprehensive specialized hospital, Ethiopia 2022.

**Method:**

Institutional-based retrospective cross-sectional study design was conducted from January1, 2019 to December 31, 2021 at University of Gondar comprehensive specialized hospital. All 200 children were included in the study and Data were extracted through structured check-list. The data were entered using EPI DATA version 4.6 and exported to STATA version 14.0 for data analysis.

**Results:**

From the total of two hundred pediatric patients 44% had delayed diagnosis and the median delay diagnosis was 68 days. Rural residence (AOR = 1.96; 95%CI = 1.08–3.58), absence of health insurance (AOR = 2.21; 95%CI = 1.21–4.04), Hodgkin lymphoma (AOR = 9.36; 95%CI = 2.1-41.72), Retinoblastoma (AOR = 4.09; 95%CI = 1.29–13.02), no referral (AOR = 6.3; 95%CI = 2.15–18.55) and absence of comorbid disease (AOR = 2.14; 95%CI = 1.17–3.94) were significant factors associated with delay in diagnosis.

**Conclusion and recommendation:**

Delayed in diagnosis of childhood cancer was relatively lower than previous studies and most influenced by the child’s residency, health insurance, type of cancer and comorbid disease. Thus; every effort should be made to promote public and parental understanding of childhood cancer, promote health insurance and referral.

## Background

Delay in diagnosis of cancer (DDC) is defined as the interval between the onset of symptoms and confirmed diagnosis of cancer [1]. It is one of the major health problem that contribute to decreased survival rates of children in under developed nations which is associated to non-specific symptoms of childhood cancer, nature of tumor and other health care system factors [2].

It is the leading cause of disease-related death in children in developing countries like Ethiopia and remains an important public health concern because of its great physical and psychological impact on the affected children and their families [3]. Many possible risk factors for development of cancer in children and adolescents have been investigated [3]. However, the causes of childhood cancer are mostly unknown. Currently, early diagnosis followed by effective treatment is an essential approach for control of the public health burden due to childhood cancer. Appropriate early diagnosis and treatment require primary care physicians and parents to be aware of early symptoms of childhood malignancies. Public and professional education can be effective in eliminating disparities in cancer survival [4]. In spite of these recommendations, pediatric cancer diagnosis delays have not received as much attention as cancers in adults in Ethiopia.

Late diagnosis and treatment of children with cancer had a serious and life treating effect in the future of their life. The survival chance of child with cancer in Low and Middle income countries (LMICs), is still a major problem and is less than 30%, whereas 80% in developed countries in which the variation might be related to delay in diagnosis [5]. According to the Ethiopian ministry of health national childhood and adolescent cancer control plan (2019–2023), it is estimated that around 6,000 childhood cancer cases are registered every year, and 80% die from their conditions which shows extremely low cure rate for childhood cancer is mainly attributed to the country’s inability to provide cancer treatment [6]. The plan also stated that most childhood cancers are hard to recognize, patients arrive at health centers with an advanced level of the disease, and this has also contributed to the high mortality rate [6].

Causes of delays may be patient and/or parent, nature of tumor, and healthcare associated factors. Studies indicated that factors that may be related to diagnosis delay are the child’s age at diagnosis, parent level of education, type of cancer, presentation of symptoms, tumor site, and first medical specialty consulted [7].

Most of the studies addressing delay were done in high-income countries[8]. A study conducted in South Africa revealed considerable delay in diagnosing of childhood cancer mostly physician delay [9]. Absences of health insurance in Kenya was associated with delay and abandonment of treatment [10]. Whereas, A study conducted in Egypt, indicated that patient aged (< 5 years), lower parental education, socioeconomic status, malignancy type and tumor site were affected significantly the time for diagnosis of childhood cancer [11].

In Ethiopia, few studies have been conducted on delay in diagnosis of childhood cancer. A study conducted on Clinical Presentation of Retinoblastoma at Jimma University Medical Center Pediatric Oncology Unit, showed that most of 24 (75%) of the patients presented with advanced stage (proptosis and fungating orbital mass) of the disease [12]. This indicates that the management of cancer in children becomes worse with poor prognosis due to long diagnosis delay time [13].

The most effective strategy to reduce the burden of cancer in children and improve outcomes is to focus on a prompt, timely diagnosis followed by effective, evidence-based therapy with simple supportive care [9]. Even though national attempts have been tried on early diagnosis of childhood cancer to improve the quality of life and survival rate, there is still a big problem of delay in diagnosis due to different factors and the mortality rate is greater than 80% in Ethiopia [14].

Few studies have been published on prevalence and associated factors of diagnosis delays in childhood cancer in Ethiopia. To our knowledge, no study was done on this topic in the study area. Therefore, this study aimed to assess prevalence and associated factors of delay in diagnosis of childhood cancer at university of Gondar specialized hospital pediatric oncology ward.

## Methods

### Study design and period

Institutional- based retrospective cross-sectional study was conducted from January1, 2019 to December 31, 2021.

### Study area

The study area was university of Gondar comprehensive specialized hospital located at Central North Gondar Zone, Amhara Regional state, Ethiopia. The hospital is found 750 km far from Addis Ababa, capital city of Ethiopia and 171 km away from Bahir Dar city of Amhara regional state. It is the largest hospital in Central-North Gondar zone serving for more than 5 million people per year [15]. It is a multidisciplinary comprehensive specialized hospital with 550 beds. University of Gondar comprehensive specialized hospital is the only hospital that gives functional pediatric oncology service in the region which has 01 oncologist pediatrician, 11 BSC comprehensive nurses, and temporary resident and intern doctors in the ward. The unit has 35 beds but no separate radiology, pathology and physiotherapy units.

### Study population

All registered children diagnosed with cancer at university of Gondar comprehensive specialized hospital admitted to pediatric oncology ward from January 1, 2019 to December 31, 2021.

### Inclusion criteria

All medical records of children diagnosed with cancer aged ≤ 18 years in pediatrics cancer unit at university of Gondar comprehensive specialized hospital from January1, 2019 to December 31, 2021.

### Sample size techniques and sampling procedure

Since the total number of the population under investigation was small (200), Census method was applied (all registered children diagnosed with cancer at university of Gondar comprehensive specialized hospital admitted to pediatric oncology ward included as study population). According to pediatric cancer patients’ report from the registration book, on average 5.6 children have been visited pediatric oncology ward per month and sixty seven per year. Therefore, the study population size was all 200 medical charts of children diagnosed with cancer who were registered from January 1, 2019 to December 31, 2021.

### Variables of the study

**Dependent variable**: Delay in Diagnosis.

#### Independent variables

**Socio-demographic characteristics of child** (sex, age, residence, health insurance).

**Clinical characteristics (**sign/symptom, type of malignancy, comorbid illness**)**.

**Health care associated characteristics** (first visited facility, hospital level, referral, source for referral, medical specialty of care provider).

### Operational definition

#### Delay in diagnosis

The time interval between cancer manifestations detected to confirmed diagnosis (if median diagnosis time ≥ 90 days it is delayed) [16].

#### Patient delayed diagnosis

The time between the onset of symptoms & signs detected by the patients to first health care visit (if median time ≥ 50days) [16].

#### Physician delayed diagnosis

the time interval from the first health care provider contact to confirmed cancer diagnosis (if median time ≥ 32days) [16].

### Data collection tool and procedure

The data were collected by using structured checklist which was adapted from different literatures [11, 16, 17]. The tool was prepared in English version and the data were extracted through reviewing patient’ medical chart by trained data collectors. Two BSc clinical nurses and one BSc nurse (ward head) supervisor were participated for data collection.

### Data Quality Control

To assure the data quality, chart review was done on 5%( 10) of the study population to test the checklists’ structure, completeness and essential modifications were made accordingly at University of Gondar comprehensive specialized hospital. Cronbach alpha (0.75) was measured to check reliability of the tool. Face tool validation checked by experts; training for data collectors and supervisor was given prior to data collection for half day.

### Data processing and analysis

The data were checked by the principal investigator on a daily basis during data collection for completeness, and consistencies. Collected Data were then coded, entered to EPI data V4.6, cleaned and analyzed using STATA version 14.00 Software. Descriptive analysis was used to describe the frequencies and percentages of the variables in the study. The strength of association was measured using adjusted odds ratio and 95% confidence interval. Binary logistic regression analysis was used to test associations between independent variables and the dependent variable. Variables with P-value < 0.25 in bi-variable analysis were included to the multivariable logistic regression model. Finally, variables with P-value < 0.05 were considered as potential determinants of delayed diagnosis among pediatric cancer patients. The Hosmer-Lemeshow logistic regression model was fitted at (x^2^ = 10.36; p = 0.241).

## Results

### Socio-demographic characteristics of study participants

A total of 200 participants were included in the study with newly diagnosed malignancy making response rate of 100%. About 131 (65.5%) of the study participants were males. The median and interquartile range (IQR) of participants’ age was 7 ± 6 years in which most of them were found in the age category of 5–10 years. Majority 124(62%) of the participants were urban residents. 88(44%) of respondents had health insurance (Table 1).

**Table 1:** Socio-demographic characteristics of study participants with cancer in UOGCSH, Gondar, North West, Ethiopia, 2022(N = 200)

Variables		
	Frequency (N)	Percent (%)
**Sex**		
Male	131	65.5%
Female	69	34.5%
**Residence**		
Rural	76	38%
Urban	124	62%
**Health insurance**		
Yes	88	44%
No	112	56%
**Age**		
< 5 years	64	32%
5–10 years	87	43.5%
10-≤18 years	49	24.5%

### Clinical/Health care-related characteristics of participants

Majority of pediatric patients’ first contact with health care providers 162(81%) cases had seen in hospital, 42(21.0%) of children had referral paper and most of them were from specialized hospital 87(53.7%) (Table 2).

**Table 2:** Health care system literacy of caregivers among children with cancer in UOGCSH, Gondar, North West, Ethiopia, 2022(N = 200)

Variables	Delayed diagnosis	
	Yes	No
**First visited facility**		
Health center	11 (5.5%)	10 (5%)
Hospital	70 (35%)	92 (46%)
Private clinic	7 (3.5%)	10 (5%)
**Referral**	42 (21%)	158 (79%)
**Source of referral**		
Health center	3 (7.14%)	10 (23.81%)
Hospital	1 (2.38%)	17 (40.48%)
Private clinic	4 (9.52%)	7 (16.67%)
**Hospital level**		
Primary hospital	17 (10.49%)	30 (18.52%)
General hospital	15 (9.26%)	13 (8.02%)
Specialized hospital	37 (22.84%)	50 (30.86%)

### Health care providers’ specialty and first contact evaluation at cancer treatment center

**Fig. 1:**
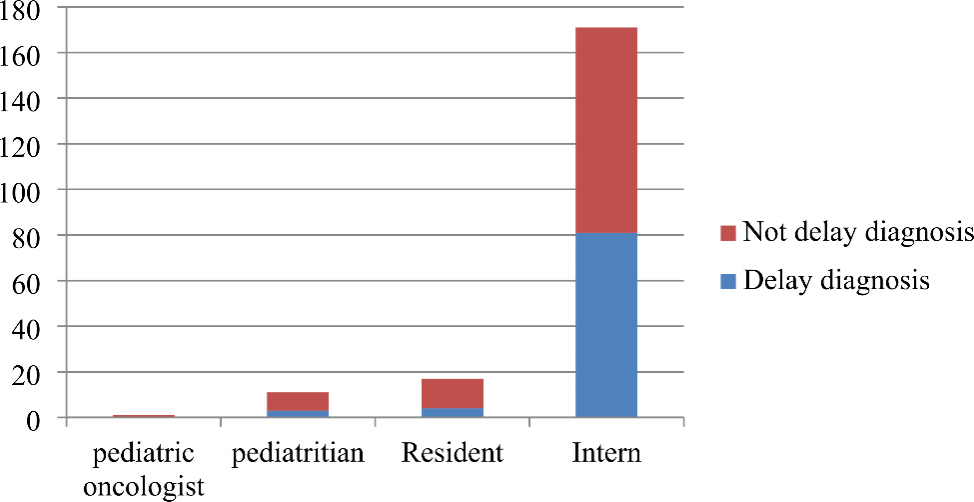
Health care providers’ specialty and first contact evaluation at cancer treatment center at University of Gondar Comprehensive Specialized Hospital, North West, Ethiopia, 2022 (N = 200)

Most patients 171(85%) were initially evaluated by intern doctors, 17(8.5%) evaluated by resident physicians. Weight loss 63(31.5%), pain 46(23%), abdominal mass 42(21%) and eye related sign/symptoms 12(6%) were presenting common signs/symptoms among children with cancer. Around 85(42.50%) of children with cancer had comorbid disease while the rest had not (Fig. 1).

### Delay in diagnosis of cancer among children age ≤ 18 years admitted at oncology ward

From the total of 200 patients, 88 (44%) [CI; 37-51%] had delayed diagnosis in which the majority of the delayed in diagnosis (50%) was contributed by patients’ delay.

### Delay in diagnosis of cancer among children depened on the type of cancer

**Fig. 2:**
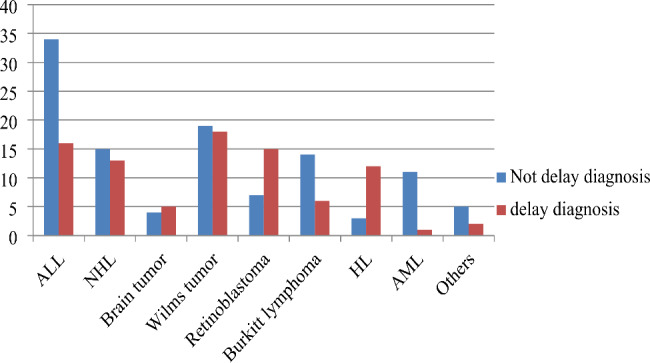
Delay in diagnosis of cancer among children based on type of cancer in University of Gondar comprehensive specialized hospital, Gondar, North West Ethiopia, 2022 (N = 200)

The most common malignancy were ALL 50 (25%) followed by Wilms tumor 37(18.5%) and NHL 28(14%). Patients who had Wilms tumor were more delayed to be diagnosed (Fig. 2).

### Factors associated with delay in diagnosis of cancer among children

In the bi-variable analysis, sex, referral, residence, health insurance, comorbid disease, HL, NHL, Wilms tumor and Retinoblastoma were factors that had a p-value < 0.25. Variables that had a p-value of < 0.25 in the bi-variable analysis were further analyzed using multivariable logistic regression. The results of this analysis showed that the area of residence, health insurance, referral, Hodgkin lymphoma, Retinoblastoma and comorbid disease were found to be significantly associated with delayed diagnosis of cancer in children.

The odds of those children living in rural residence were 1.96 (AOR = 1.96; 95%CI = 1.08–3.58) times more likely to get diagnose delayed relative to urban residents. Similarly, the odds of children who have no health insurance were 2 times (AOR = 2.21; 95%CI 1.21–4.04) more likely to be diagnosed delay as with compared to those who have health insurance. Moreover, the odds of children who had not comorbid disease were 2 times (AOR = 2.1; 95%CI = 1.01–4.32) more likely to get delayed.

Children having no referral had odds of 6.3 (AOR = 6.3; 95%CI = 2.15–18.55) times more likely delay in diagnosis than children who had referral. Those who had Hodgkin lymphoma had odds of 9.4 (AOR = 9.36; 95%CI = 2.1- 41.72) times more likely delay in diagnosis than who had Acute Lymphoblastic leukemia and those who had Retinoblastoma were 4.1 (AOR = 4.09; 95%CI = 1.29–13.02) times more likely delayed diagnosis than who had Acute Lymphoblastic leukemia (Table 3).

**Table 3:** Factors associated with delay in diagnosis of cancer among children aged ≤ 18 years (UOGCSH), 2022(N = 200)

Variables	Category	Delay in Diagnosis		COR (95%CI)	AOR (95%CI)
		Yes	No		
Sex	Female	25(36.23%)	44(63.77%)	0.61(0.34–1.12)*	1.14(0.13–9.97)
	Male	63(48.09%)	68(51.91%)	1	1
Residence	Rural	42(55.26%)	34(44.74%)	2.09(1.17–3.74)*	1.96(1.08–3.58)**
	Urban	46(37.1%)	78(62.9%)	1	1
Health insurance	Yes	30(34.09%)	58(65.91%)	1	1
	No	58(51.79%)	54(48.21%)	2.1(1.17–3.69)*	2.21(1.21–4.04)**
Comorbid disease	Yes	30(35.29%)	55(64.71%)	1	1
	No	58(50.43%)	57(49.57%)	1.9(1.05–3.32)*	2.1(1.01–4.32)**
Cancer type	HL	12(80%)	3(20%)	9(2.23–36.33)*	9.4(2.1-41.72)**
	NHL	13(46.43%)	15(53.57%)	1.84(0.71–4.77)*	1.54(0.54–4.38)
	Wilms	19(51.35%)	18(48.65%)	2.24(0.93–5.39)*	1.53(0.63–4.27)
	Retinoblastoma	14(68.18%)	8(31.82%)	3.94(1.38–11.25)*	4.1(1.29–13.03)**
	ALL	16(32%)	34(68%)	1	1
Referral	Yes	9(21.43%)	33(78.57%)	1	1
	No	79(50%)	79(50%)	3.7(1.65–8.16)*	6.3(2.15–18.55)**

N.B; *=p-value < 0.25 and **=p-value < 0.05

## Discussion

The aim of this study was to identify delay in diagnosis and associated factors of pediatric cancer patients at university of Gondar Comprehensive Specialized Hospital. The overall current finding revealed that 44% of participants had total delay in diagnosis. This finding is lower than study conducted in Argentina(63.5%) and Brazil(55%) [18, 19]. This discrepancy might be due to differences in sample size and included cancer type in the study.

The current finding is also lower than the study conducted in Mekele (69.6%) and Bangladesh (70%) [16, 20]. The difference might be due to shorter diagnosis time by physician in current study that enhances timely decision on pediatric cancer diagnosis, study period, cut off point in diagnosis delay and difference in socio-demographic characteristics of the study participants.

Residence, referral, health insurance, comorbid disease, Retinoblastoma and Hodgkin lymphoma cancers were significant factors that contribute for diagnosis delay. Children with Retinoblastoma had odds of 4.1 times more likely delay to be diagnosed than children having acute lymphoblastic leukemia keeping other variables constant. This finding is similar with that of the study done in Jimma comprehensive specialized hospital and Nigeria [12, 21]. This similarity might be due to a high risk of the cancer being missed on examination, either because tumors are located in the anterior retina, because the examination has not been done correctly, or because the child is unable to co-operate with the exam [22].

Likewise, in the current finding children having Hodgkin lymphoma had odds of 9.4 times more likely delay to be diagnosed than children having acute lymphoblastic leukemia keeping other variables constant. This might be the fact that some tests use specialized equipment or need specially trained experts to find out the exact genetic make-up of lymphoma cells. The samples might need to be sent to a different laboratory and can take several times [23].

From our Findings the risk of increased delay for children who were living in rural area was 1.96 times more likely delay than urban residence keeping other variables constant. the current finding is comparable with the study from Mekele Ayder Hospital [16]. This is due to rural areas might be far from cancer treatment center, transport inaccessibility and parents’ low health seeking behavior.

Children who have no health insurance had odds 2 times more likely to be diagnosed delay as with compared to those who have health insurance. This is similar with a study done in Kenya and Mekele [10, 16]. Where have no insurance increased the risk of delayed in diagnosis. Regarding the referral system, Children who had no referral had odds of 6.3 times more likely delay in diagnosis of childhood cancer than children who had referral. This finding is in line with the study conducted in Ibadan, Nigeria [21].

Moreover, Children who have no comorbid disease were 2 times more likely to be diagnosed delay as with compared to those who have comorbid disease keeping other variables constant. This might be due to patients having comorbid disease seeking more health care and may shorten delay in diagnosis of childhood cancer.

### Limitation of the study

The study couldn’t incorporate parental socio-demographic characteristics and opinions because of the nature of study design and secondary data usage. It may not be representative of the whole country cases as it is a single institutional study. It is also difficult to determine strong cause and effect relationships between dependent and independent variables since it is a cross-sectional study.

## Conclusion and recommendation

Although delays in diagnosis of cancer among children were relatively low as compared to other studies, the prevalence of delay in diagnosis of cancer among children remains prevalent in the study area. Rural residence, absence of health insurance, Hodgkin lymphoma, Retinoblastoma, no referral and absence of comorbid were significant factors associated with delay in diagnosis of childhood cancer. Therefore, it is recommended to conduct either large-scale community-based or qualitative study to address those factors that hinder early diagnosis of cancer in children. It is also better to expand referral linkage, promote health insurance to patients and children shall be evaluated more by senior health care providers.

## Data Availability

Data generated/ analyzed during this study are found from the corresponding author.
